# Delivery of Compassionate Mental Health Care in a Digital Technology–Driven Age: Scoping Review

**DOI:** 10.2196/16263

**Published:** 2020-03-06

**Authors:** Jessica Kemp, Timothy Zhang, Fiona Inglis, David Wiljer, Sanjeev Sockalingam, Allison Crawford, Brian Lo, Rebecca Charow, Mikayla Munnery, Shuranjeet Singh Takhar, Gillian Strudwick

**Affiliations:** 1 Faculty of Science University of Waterloo Waterloo, ON Canada; 2 Information Management Group Centre for Addiction and Mental Health Toronto, ON Canada; 3 Office of Education Centre for Addiction and Mental Health Toronto, ON Canada; 4 Education Technology and Innovation University Health Network Toronto, ON Canada; 5 Institute of Health Policy, Management and Evaluation University of Toronto Toronto, ON Canada; 6 Department of Psychiatry University of Toronto Toronto, ON Canada; 7 Campbell Family Mental Health Research Institute Centre for Addiction and Mental Health Toronto, ON Canada

**Keywords:** compassion, mental health, medical informatics, psychiatry, health information technology, nursing informatics

## Abstract

**Background:**

Compassion is a vital component to the achievement of positive health outcomes, particularly in mental health care. The rise of digital technologies may influence the delivery of compassionate care, and thus this relationship between compassion and digital health care needs to be better understood.

**Objective:**

This scoping review aimed to identify existing digital technologies being used by patients and health professionals in the delivery of mental health care, understand how digital technologies are being used in the delivery of compassionate mental health care, and determine the facilitators of and barriers to digital technology use among patients and health professionals in the delivery of compassionate mental health care.

**Methods:**

We conducted this scoping review through a search of Cumulative Index to Nursing and Allied Health Literature, Medical Literature Analysis and Retrieval System Online (MEDLINE), MEDLINE In-Process and EPub Ahead of Print, PsycINFO, and Web of Science for articles published from 1990 to 2019.

**Results:**

Of the 4472 articles screened, 37 articles were included for data extraction. Telemedicine was the most widely used technology by mental health professionals. Digital technologies were described as facilitating compassionate care and were classified using a conceptual model to identify each digital intersection with compassionate care. Facilitators of and barriers to providing compassionate care through digital technology were identified, including increased safety for providers, health care professional perceptions and abilities, and the use of *picture-in-picture* feedback to evaluate social cues.

**Conclusions:**

Implementing digital technology into mental health care can improve the current delivery of compassionate care and create novel ways to provide compassion. However, as this is a new area of study, mental health professionals and organizations alike should be mindful that compassionate human-centered care is maintained in the delivery of digital health care. Future research could develop tools to facilitate and evaluate the enactment of compassion within digital health care.

## Introduction

### Background

The use of digital technology in mental health care delivery has increased significantly in recent years [1-5]. Improved patient access to mental health services is a common metric used to endorse the use of technology in mental health care through technologies such as telepsychiatry [6]. Owing to the emerging uptake and expansion of digital technologies into a variety of traditional and nontraditional care settings, such as in-home use of digital technologies or the provision of care in virtual environments, there exists a greater need to understand best practices surrounding digital technology use to ensure quality patient-centered care is delivered through these modalities [7-11]. Fostering the delivery of compassionate care has been identified as an important need because compassion has been shown to positively influence the experience of both patients and health professionals alike [7]. Without an adequate understanding of the best practices and uses of digital technology for the delivery of compassionate mental health care, these technologies may detract from compassionate care and hinder health professional-patient relationships, which are of great importance in the context of mental health care. However, when employed appropriately, these same technologies may facilitate and strengthen compassionate mental health care and create new means for relationships between mental health professionals and patients [12].

### Compassion in Health Care

*Compassion* encompasses a wide array of meanings [13,14]. The working definition of compassion used for the purpose of this review defines five dimensions of compassion: (1) awareness of another’s experience of suffering or need, (2) feeling *moved*, (3) recognizing this feeling as a response to the other’s need, (4) making a judgement that the other is suffering, and (5) engaging in a behavior in an attempt to alleviate the suffering [15]. The importance of compassion in mental health care is central; for many patients, receiving compassionate care throughout the process of diagnosis, treatment, and recovery can improve their perceived quality of care [16-18]. When compassion is present in mental health care settings, there can be a greater therapeutic alliance (the quality of the relationship between provider and patient), increased openness of the patient which improves health professionals’ understanding of a patient’s experiences, and greater experiences of empathy as part of the health professional-patient relationship, ultimately supporting patient-centered care [17-22].

### Delivery of Compassionate Mental Health Care

The delivery of compassionate mental health care can take many forms, with the foundation being to remain patient focused, establishing interactions based on trust, and ensuring physical and emotional safety [23]. Compassionate care may be subjectively experienced; however, the literature suggests that it is commonly delivered by providing safe and comfortable spaces for health professional-patient interactions and is rooted in an understanding by health professionals of the lived experiences of patients [19,24,25]. It is important to recognize that not all patients will experience the feeling of compassion or build a compassionate relationship in the same way; however, digital technology has the potential to meet a wide range of patient needs and provide more personalized care due to the adaptability of technology [26].

### Digital Technology Use and Compassionate Mental Health Care

Many digital technologies are currently being used in mental health care contexts, including (but not limited to) mobile apps, patient portals, electronic health records (EHRs), instant messaging, telemedicine, and virtual reality [27]. This review arose out of the motivation to understand what is known about the suitability of these technologies for facilitating or enhancing compassionate care and whether any evidence can guide best practices for use.

### Purpose

The purpose of this review was to identify the ways in which compassionate care can be delivered in mental health care through and with the use of digital technologies, as well as across the continuum of mental health care settings and processes. To develop an understanding of the intersection between digital technology and compassionate mental health care, this review examines 3 research questions (RQs):

1. What existing digital technologies are most commonly used among patients and/or health professionals in the delivery of mental health care?
2. How are existing digital technologies being used among patients/health professionals in the delivery of compassionate mental health care?
3. What are the perceived facilitators of and barriers to using digital technology among patients and/or health professionals to deliver compassionate mental health care?

## Methods

### Overview

**Figure 1:**
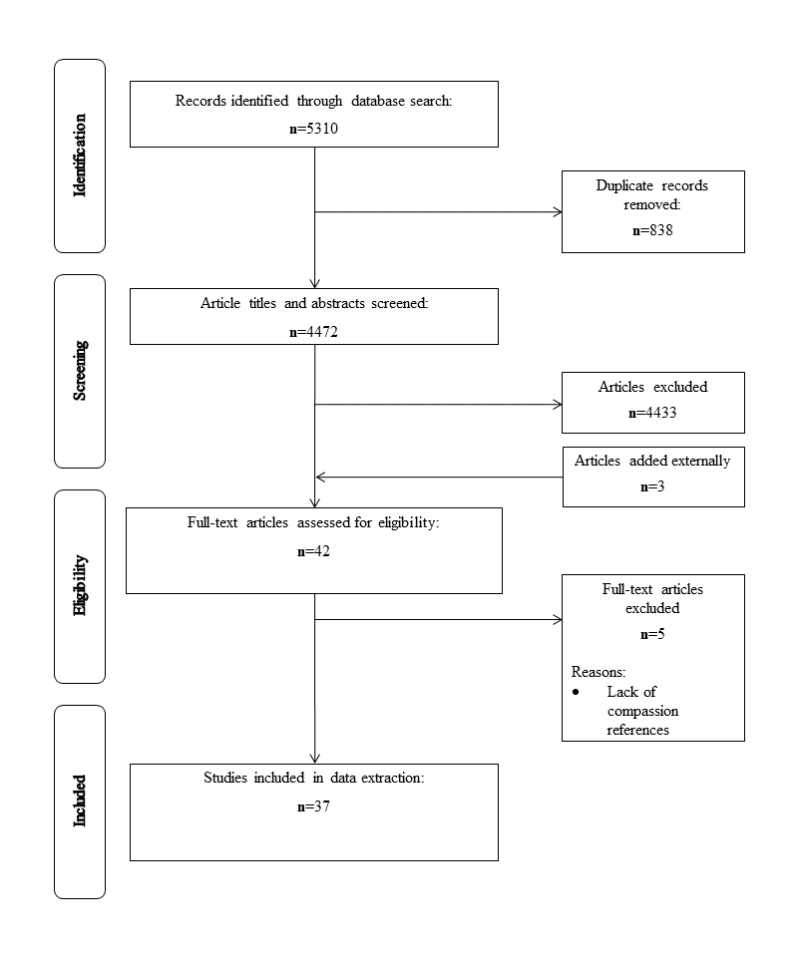
Preferred Reporting Items for Systematic Review and Meta-Analysis flow diagram of scoping review results.

This review was conducted following the methodological framework for scoping review studies proposed by Arksey and O’Malley [28] and refined by Levac et al [29]. To illustrate the scoping review process, the Preferred Reporting Items for Systematic Review and Meta-Analysis (PRISMA) diagram [30], shown in Figure 1, was used as well as the PRISMA-scoping review checklist which outlines the key milestones of a scoping review [31] (Multimedia Appendix 1). A detailed protocol for this scoping review titled *Delivery of Compassionate Mental Health Care in a Digital Technology-Driven Age: Protocol for a scoping review* was published in *BMJ Open* [32]. The following sections provide a brief overview of the methodology utilized in this scoping review.

### Stage 1: Identifying the Research Questions

For the purpose of this scoping review, the aforementioned RQs were identified to better understand the delivery of compassionate mental health care through and with the use of digital technology.

### Stage 2: Identifying Relevant Studies

All types of research studies including quantitative, qualitative, observational, and literature reviews from any country of origin published in English from 1990 to 2019 were included. Given the nature of the topic being investigated, grey literature was not included. All areas of mental health care, diagnoses, digital technologies used, and age groups were included. Studies were selected if they addressed at least 1 of the 3 RQs and involved the use of digital technology in mental health care in relation to compassionate care. As compassion is a difficult concept to define, the working definition of compassion described earlier was used to guide the identification of eligible articles involving compassionate care. Although all types of digital technology were eligible, some imaging and measurement technologies such as those intended to measure emotion, blood pressure, or conduct body scans were excluded [32].

A total of 5 databases were searched: Cumulative Index to Nursing and Allied Health Literature, Medical Literature Analysis and Retrieval System Online (MEDLINE), MEDLINE In-Process and EPub Ahead of Print, PsycINFO, and Web of Science. A research librarian (FI) completed the search strategy and database searches. As a part of working with a librarian, extensive use of synonyms, Boolean operators, combinations of search terms, and MeSH headings were employed. The complete search strategy for MEDLINE is available in the published protocol for this scoping review [32].

### Stage 3: Study Selection

All identified articles were screened independently by 2 reviewers (TZ and AM), concluding with an interrater reliability of 99.22% agreement and a Cohen kappa of 0.59. Disagreements which could not be resolved between TZ and AM were discussed with the greater research team, as outlined in the study protocol [32]. The screening process was facilitated by Covidence (Veritas Health Innovation), a literature review streamlining software recommended by Cochrane [33].

While identifying the relevant studies for the scoping review through the screening process, the authors selected articles that either directly facilitated the delivery of compassionate care or prepared for the delivery of compassionate care while addressing 1 or more of the 5 dimensions of compassion. It is important to note that compassion was not always explicitly brought up in some articles and the professional judgement of the authors had to be used to identify appropriate studies. Upon further research and completion of data extraction, it was evident that there was a greater divide among the relevant studies. The authors chose to use the digital intersections with compassion to further clarify the role/dimension each technology played in the delivery of compassionate care.

### Stage 4: Data Items and Data Collection Process

During the process of data extraction, the following article summary information was charted: title, authors, year of publication, country of origin, research design, RQs addressed, and answers to the applicable RQs. Data were charted using Microsoft Excel 2010. The data extraction table is available upon request from the corresponding author.

### Stage 5: Synthesizing and Reporting the Results

Both quantitative and qualitative methods were used to analyze the results of the RQs. A descriptive quantitative analysis (descriptive statistics) was used for RQ1, and a qualitative content analysis was used for RQ2 and RQ3. To understand what existing digital technologies are most commonly used among patients and health professionals in the delivery of mental health care, the results of RQ1 were organized using the World Health Organization’s (WHO) classification of digital health interventions v1.0 [34]. This classification system organizes digital technologies used in health care based on the user of each intervention.

### Stage 6: Consultation

The consultation phase for this review was completed through discussions with mental health and digital health researchers, mental health professionals, and various health care professionals in Ontario, Canada, selected through the Associated Medical Services (AMS) health care community. More specifically, these stakeholders were consulted at Waypoint Centre for Mental Health Care, the Centre for Addiction and Mental Health, the University of Toronto, and Western University. The consultation process was important for the organization of results and to ensure the strategies used for knowledge translation were appropriate. These discussions also supported the identification of important topics to include in the Discussion section of this paper.

## Results

### Search Results

A total of 37 articles were included in the final review. Details regarding the screening process are described in Figure 1.

### Study Characteristics

Table 1 describes the characteristics of the studies included in this review. Studies were identified from 7 countries with 57% (21/37) of these publications originating from the United States. Given the novelty of digital technology use in mental health care, 51% (19/37) of articles were published between 2016 and January 2019. A research focus on a specific mental health diagnosis was uncommon in the selected articles; only 27% (10/37) of the articles were related to a specific diagnosis. Articles that did not specify a mental health diagnosis, and rather addressed mental health care as a single entity or did not specify the diagnoses of patients, were categorized as *unspecified*. Table 1 also includes the methods that were used in the identified articles.

**Table 1:** Study characteristics.

Article characteristics		Value (N=37), n (%)	References
**Country of publication**			
	United States	21 (57)	[24,35-54]
	United Kingdom	8 (22)	[19,20,55-60]
	Australia	4 (11)	[61-64]
	Canada	1 (3)	[65]
	China	1 (3)	[66]
	Israel	1 (3)	[26]
	The Netherlands	1 (3)	[67]
**Research method**			
	Literature review	16 (43)	[20,26,37,40,42,44-48,50,54,62-64,67]
	Questionnaire/survey	8 (22)	[24,49,52,53,56,61,65,66]
	Mixed method	5 (14)	[35,55,57-59]
	Semistructured interview	4 (11)	[38,41,43,60]
	Other^a^	4 (11)	[19,36,39,51]
**Year of publication**			
	2016-2019	19 (51)	[24,38,40,41,47-55,57,59,61,64-66]
	2010-2015	13 (35)	[19,20,26,35,37,43-45,56,58,60,62,67]
	2000-2009	5 (14)	[36,39,43,46,63]
**Mental health diagnosis^b^**			
	Unspecified	27 (73)	[20,24,26,35,37-48,50,54,55,57,59,62-67]
	Schizophrenia and psychosis	3 (8)	[51,53,61]
	Anxiety and depression	3 (8)	[19,52,58]
	Trauma and stress disorder	2 (5)	[36,49]
	Alzheimer and dementia	2 (5)	[56,60]
	Addictions/substance use	0 (0)	—^c^
	Developmental disabilities	0 (0)	—
	Problem gambling	0 (0)	—
	Mood and personality disorders	0 (0)	—

^a^Other research methods include group therapy sessions and personal essays written by health professionals.

^b^Categories of mental health diagnosis based on the Centre for Addiction and Mental Health’s Mental Illness and Addiction Index [68].

^c^No articles were identified.

### Research Question 1: Digital Technology Use in Mental Health Care

Of the 37 articles, 15 [19,24,39,41,42, 45,46,48,51,52,57,58, 64-66] and 22 [20,26,35-38,40,43,44,47,49,50,53-56,59-63,67] articles were specific to digital technology usage by patients and health professionals, respectively. Patient and health professional digital health interventions were then divided into detailed categories, as shown in Table 2. On-demand information services were the most common digital technology used by patients (eg, educational resources), including websites [52,65], mobile phones [51], and apps [57,64,66]. Targeted patient communication technologies [19,24,39,45] had the second highest frequency among patients. This category comprises personalized information that is delivered to individuals or groups of patients from health professionals and can be unidirectional (a message can only be sent by the health professional; patients do not have the option to reply) or bidirectional (patients can reply to messages from the health professional) [69]. It is important to note that the definition of this category proposed by the WHO is only limited to unidirectional communication from the health professional [34]. Some examples of targeted patient communication observed in this review included humanoid animated agents (computational artifacts used to develop human-like relationships with patients through the development of trust, rapport, and therapeutic alliance [45]) used for the purpose of computerized cognitive behavioral therapy (CBT) [45], email communication [39], and websites [24]. In all, 2 articles included examples of patient-to-patient communication via digital technology, including online peer-support groups and chatrooms [42,46]. Personal health tracking interventions included patient portals and EHRs, with the primary function of self-monitoring [41,48]. Untargeted patient communication (generalized communications distributed to a large patient population in which all recipients receive identical messages [34]) was the least common digital health intervention, consisting of a computerized CBT program with a singular set of responses generated for all users [58].

There were 22 cases of digital health interventions being used by health professionals including telemedicine, health professional training, and patient health records. Telemedicine (providing health care from a distance through the use of technology [34]) made up 78% (17/22) of all types of digital health interventions used by health professionals [20,26,35,36,38,40,44,47,50,54-56,59,62,63,67]. In this review, telemedicine was observed through the use of videoconferencing [20,26,36,38,40,44,50,54,62,63,67], apps [47], telephone communication [55], gaming [35,56], and virtual reality [20,59] to provide patient care. Digital technology was also commonly used among health professionals for training purposes through the use of virtual reality [53,60,61] and apps [49]. The last digital health intervention used among health professionals included in this review was the use of EHRs during patient appointments [43].

**Table 2:** Digital health interventions.

The World Health Organization classification of digital health interventions^a^		Frequency	References
**1.0 Patients**		**15**	**—^b^**
	1.1 Targeted patient communication	4	[19,24,39,45]
	1.2 Untargeted patient communication	1	[58]
	1.3 Patient to patient communication	2	[42,46]
	1.4 Personal health tracking	2	[41,48]
	1.5 Citizen-based reporting	0	—
	1.6 On-demand information services to patients	6	[51,52,57,64-66]
	1.7 Patient financial transactions	0	—
**2.0 Health Professionals**		**22**	**—**
	2.1 Patient identification and registration	0	—
	2.2 Patient health records	1	[43]
	2.3 Health professional decision support	0	—
	2.4 Telemedicine	17	[20,26,35-38,40,44,47,50,54-56,59,62,63,67]
	2.5 Health professional communication	0	—
	2.6 Referral coordination	0	—
	2.7 Health worker activity planning and scheduling	0	—
	2.8 Health professional training	4	[49,53,60,61]
	2.9 Prescription and medication management	0	—
	2.10 Laboratory and diagnostics imaging management	0	—

^a^The WHO classification system terminology employs clients and health care providers; in the context of this review, patients and clients will be interchangeable as well as health care providers and health care professionals.

^b^No articles were identified.

### Research Question 2: Delivery of Compassionate Mental Health Care Through and With Digital Technology

**Figure 2:**
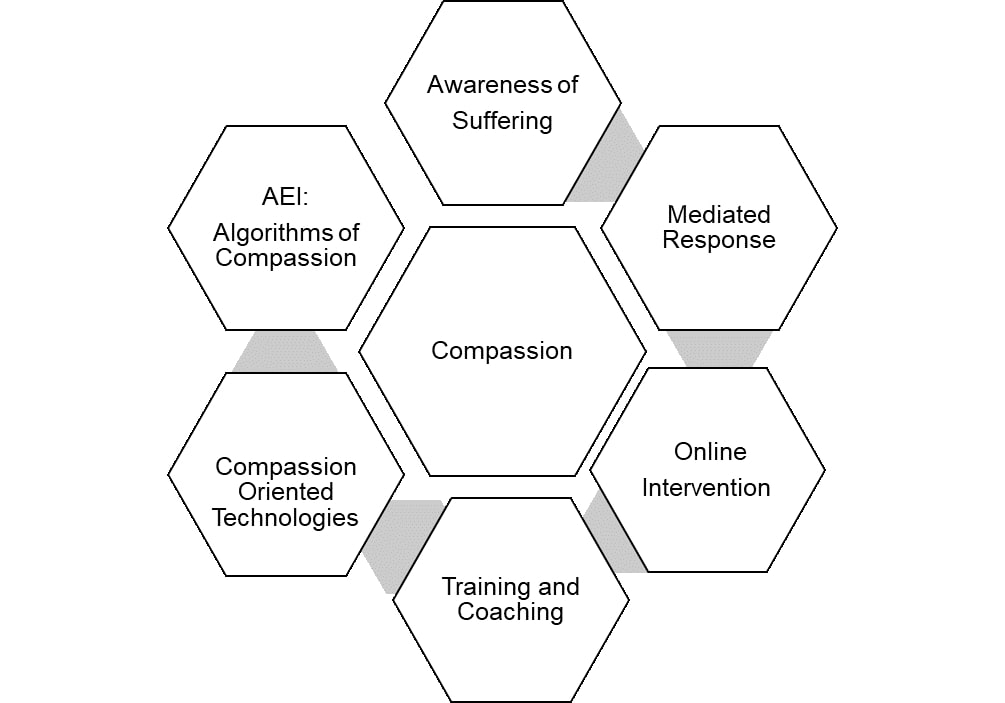
Digital intersections with compassionate care. AEI: artificial emotional intelligence.

Owing to the subjectivity of the definition of compassionate care, a conceptual model titled *Digital Intersections with Compassionate Care*, shown in Figure 2 and definitions in Table 3, from the textbook chapter *Caring in a Digital Age: Exploring the Interface of Humans and Machines in the Provision of Compassionate Healthcare* [70] was used to understand and organize the unique roles of digital technology in the delivery of compassionate mental health care. This model illustrates the intersections between the 6 main components of compassionate care and digital technology [70]. An additional category was created for the purpose of this review to account for articles that proposed digital technology use in mental health care may detract from compassionate care (Table 4).

The digital intersections (definitions are included in Table 3) addressed in this review include numerous examples of online interventions, training and coaching, compassion-oriented technologies, and artificial emotional intelligence, as shown in Table 4.

**Table 3:** Definitions of the digital intersections with compassionate care.

Digital intersection with compassionate care	Definition
Awareness of suffering	Developing an awareness of one’s suffering through the use of digital technology (ie, experiences shared via digital technology increase awareness of one’s suffering)
Mediated response	Utilizing digital technology to mediate or influence one’s response to suffering
Online intervention	Responding to suffering through an online intervention
Training and coaching	Digital tools used to increase health professional expertise or patient knowledge to ensure the delivery of compassionate care (ie, through digital storytelling, online forums, and messaging systems used to share knowledge and experiences)
Compassion-oriented technologies	Digital technologies created specifically to assist in or facilitate the delivery of compassionate care
Artificial emotional intelligence	Artificial intelligence used to facilitate compassionate interactions with patients

**Table 4:** Digital intersections with compassionate care.

Digital intersection with compassionate care	Frequency	References
Awareness of suffering	0	—^a^
Mediated response	0	—
Online intervention	11	[19,20,36,38,40,42,50,57-59,63]
Training and coaching	8	[24,49,51-53,60,65,66]
Compassion-oriented technologies	14	[26,35,37,41,44,46-48,51,54,56,62,64,67]
Artificial emotional intelligence: algorithms of compassion	1	[45]
Detractions from compassionate care	3	[39,43,55]

^a^No articles were identified.

#### Online Intervention

The development of online interventions to respond to suffering (ie, responding to suffering was a direct goal or result of the intervention) was observed in 11 articles [19,20,36,38,40,42,50, 57-59,63]; this included the use of online therapy programs [42,57,58,63], virtual reality programs to portray lived experiences [20,59], email and instant messaging to respond to patient suffering [19,20], and most commonly, the use of videoconferencing for telemedicine [20,36,38,40,50].

#### Training and Coaching

Using digital technology to provide training and coaching to increase compassion demonstrates how health professionals can leverage digital technology to better understand the suffering experienced by their patients and thus respond appropriately. Virtual reality coaching was often used for health care professionals to experience simulated positive symptoms of schizophrenia and psychosis [53,61], as well as complex difficulties experienced by patients suffering from dementia [60]. Additionally, digital training and coaching was also used by patients to learn the skills and importance of mindfulness through mood tracking, tips for overall well-being, and scheduled reminders to encourage session completion, all of which were used to respond to suffering and improve care [49,52,65,66]. One article discussed digital technology used to provide training to veterans to increase their understanding of their mental health notes made accessible to them through a patient portal, reducing misinterpretations and improving provider-patient relationships [24].

#### Compassion-Oriented Technologies

Digital technologies that were classified as *compassion-oriented technologies* based on the conceptual model shown in Figure 2 were the most commonly cited. This digital intersection with compassionate care involves technologies that support compassionate care and are used by health professionals and/or patients, including uses such as shared gaming time between health professionals and patients to facilitate bonding time [35,56], mental health apps [26,47,64], and patient portals [41,48].

#### Artificial Emotional Intelligence

Artificial emotional intelligence use was infrequently documented in the delivery of compassionate mental health care but was observed in one instance through the use of humanoid animated agents as part of a computerized CBT program [45]. A humanoid animated agent simulates a face-to-face conversation and utilizes verbal and nonverbal social cues to form human-like relationships [45].

#### Detractions From Compassionate Care

A final category was created to distinguish articles that proposed digital technologies that may detract from compassionate mental health care. A total of 3 articles [39,43,55] were included in this category and included concerns regarding the effect of nonresponses (to email and instant messages) on patients [39], as well as claims from physicians who felt that relationships equivalent to those formed in-person simply could not be achieved through the use of digital technology [55].

### Research Question 3: Facilitators of and Barriers to Compassionate Mental Health Care Delivery Through Digital Technology

All articles discussed multiple facilitators of and barriers to compassionate mental health care delivery through the use of digital technology, as shown in Table 5. Facilitators included feedback on social cues, training/education for health professionals, increased safety, multilevel participation, peer-support, improved accessibility, and optional anonymity. Barriers included limitations because of health professionals’ perceptions and abilities, impersonal automated responses, lack of social cues, effect of non-responses, group size, computer use during patient encounters, poor quality of technology, and inappropriate uses of technology at various stages of illness.

Tables 6 and 7 compare functions of digital technologies, as identified in RQ3, and the digital health interventions that facilitate each function. The criterion for each category was based on the evidence provided in the articles included as part of the review; digital health interventions were only confirmed to facilitate a function if specifically mentioned in the literature. Any facilitated functions that were not applicable to a particular digital health intervention are indicated as N/A.

**Table 5:** Facilitators of and barriers to delivery of compassionate care through digital technology.

Facilitators and barriers		Support
**Facilitators**		
	Picture-in-picture functionality	The ability to view oneself on screen while interacting with a patient via videoconferencing; can allow for the evaluation of one’s own facial expressions and response to social cues [62].
	Physical distance	Patients may feel more at ease when communicating with a health professional through technology from a distance, and it also provides the opportunity to titrate the experience of distance [44].
	Training/education	Digital technology can be used for training of health professionals as well as to convey lived experiences [53,60].
	Safe for providers	Digital technology can allow health professionals to provide care without safety concerns in settings such as prisons [38,59].
	Multilevel participation	Some digital health interventions allow users to simply observe functionalities with no mandatory participation, allowing for an easier transition into care [46].
	Social/peer-support connections	Technology connects users to people with shared experiences, creating feelings of understanding and connectedness [42,46].
	Convenience/accessibility	On-demand use and the ability to reach rural and remote areas through the use of technology [36,42,50,54,62].
	Increased privacy/anonymity	Technology can allow for increased privacy and, in some cases, complete anonymity; this may decrease feelings of judgement and reduce stigma for patients [46,64].
**Barriers**		
	Health professionals’ perceptions and abilities	Some health professionals are reluctant to integrate technology into patient care because of personal perceptions and abilities [24,44].
	Impersonal	Users may receive similar resources from apps despite varying mental health concerns [42].
	Lacking social cues	The use of email and instant messaging does not allow the user to convey or evaluate tone of voice or facial expressions [19,39,42].
	Nonresponses	Patients may feel neglected because of nonresponses to emails and/or instant messages [19].
	Group size	Online self-help groups comprising large numbers of users may decrease attentiveness to patient needs and detract from individual compassionate relationships [46].
	Use of computers during patient encounters	Obstructive positioning of computers used by health professionals during a patient encounter may lead to disengagement and distraction [43].
	Quality of technology	Issues with lagging, audio problems, and poor video quality can affect the patient experience during videoconferencing used to provide telemedicine [67].
	Stage of illness	The use of some types of digital technology may only be appropriate for specific stages of illness or mental illnesses (ie, not appropriate for a crisis situation) [51].

**Table 6:** Comparison of facilitated function and associated digital health intervention.

Digital health intervention	Facilitated function				
	Evaluation of social cues	Physical distance	Personalized care	Training for health professionals	Increased safety
Telemedicine	✓	✓	✓	✕	✓
Personal health tracking	✕	✓	✓	✕	✓
Targeted client communication	✕	✓	✕	✕	✓
Untargeted client communication	✕	✓	✕	✕	✓
Client health records	✕	✓	✓	✕	N/A^a^
On-demand information services to clients	✕	✓	✓	✕	✓
Health care provider training	✕	✓	✓	✓	N/A
Client-to-client communication	✕	✓	✓	✕	N/A

^a^Not applicable.

**Table 7:** Comparison of facilitated function and associated digital health intervention.

Digital health intervention	Facilitated function				
	Multilevel participation	Risk of nonresponses	Peer support	Accessibility	Reduced stigma
Telemedicine	N/A^a^	N/A	✓	✓	✓
Personal health tracking	✓	N/A	N/A	✓	✓
Targeted client communication	✓	✓	N/A	✓	✓
Untargeted client communication	✓	N/A	N/A	✓	✓
Client health records	N/A	N/A	N/A	✓	✓
On-demand information services to clients	✓	N/A	N/A	✓	✓
Health care provider training	N/A	N/A	N/A	✓	✓
Client-to-client communication	✓	N/A	✓	✓	✓

^a^Not applicable.

## Discussion

### Digital Technology Use in Mental Health Care

This review sought to examine the relationship between the emerging use of digital technology and its effect on the delivery of compassionate care in a mental health context through 3 RQs. Implications are discussed as follows in light of the findings.

In addition to the primary findings that technologies are widely incorporated into mental health care, with an emphasis on health care delivery methods such as telemedicine (Table 3), the majority of digital technologies examined in the identified articles were not targeted toward a particular mental health diagnosis (Table 2). This finding may be because of the fact that some interventions commonly facilitated through digital technologies are applicable to multiple mental health diagnoses. For instance, while the use of CBT has typically been associated with the treatment of anxiety and depression, existing research has established that it can also be effectively tailored to treat other anxiety disorders (eg, phobias and panic disorder), schizophrenia, trauma-related disorders, and bipolar disorders [71]. A computerized CBT intervention would thus be classified as *unspecified* because users with a wide variety of needs may be able to access support and benefits through the same platform. However, emphasis should be put on the fact that such increased reach would not be possible without the delivery medium of digital technology. Similarly, the high prevalence of telemedicine use, observed in this review (Table 3), is also used as a medium to deliver varying types of mental health care rather than standing as a tailored intervention for a specific diagnosis in itself. The relatively high representation of mental health care delivery methods as opposed to specifically tailored mental health interventions for a diagnosis in relation to compassionate care may be an indication of the current infancy of the state of this area. Future research will be required to understand if the delivery of compassionate care through digital technologies varies depending on the mental health diagnosis of patients. Further, future research methodologies should include economic analysis to understand the return on investment of delivering compassionate care between mental health treatment needs.

### Digital Technologies Enabling Compassionate Care

The evolution of digital technologies is fueling the emergence of new types of health interventions. Although the decrease of the *in-person* experience may have been associated with a reduction in compassionate care [39,43,55], there are instances where a long-distance delivery of mental health care provides an improved experience for both the health care professional and patient. This review was able to substantiate that compassion is often a core aspect of digital health delivery. In fact, these new modes of intervention enable novel enactments of compassion and means to teach or train health care professionals to provide compassionate health care which would not be previously possible without digital technology. For example, for individuals requiring mental health care in correctional facility settings, escorted transportation to a satellite care site or conducting care in a monitored, secure meeting area with physical barriers may hinder the ability to build a compassionate relationship [38]. Leveraging telemedicine in situations such as this can not only cut down on resource use but also provide a more comfortable environment for both the patient and the health care professional, without which it would be difficult to deliver compassionate care [38].

In addition to areas of opportunity for improved patient experiences, emerging tools are also enhancing health professional education in fostering the delivery of compassionate care in practice. For instance, Ozelie et al present an immersive virtual reality system which offers learning through shared experiences by providing insight into the experience of a person with schizophrenia through simulated hallucinations [53]. This initiative is greatly in line with existing research that demonstrates such access to lived experiences is a highly valued resource, as shared experiences are inherently different from simply speaking or hearing about the experiences of persons with mental illness [72]. Lived experience is foundational to building relationships with others in recovery, particularly in peer-delivered services [72]. Vividly experiencing even a small portion of their patients’ experiences can allow health care professionals to better understand the patient perspective, contributing to their awareness of another’s experience of suffering or need.

Concurrently, the use of virtual reality also potentially presents itself as a natural advancement in telemedicine. Moving beyond the limitations of a 2D computer screen, virtual reality can allow for a more *in-person* experience while still capitalizing on the benefits of long-distance care [12,20,37,61]. However, unique considerations in the delivery or enablement of compassionate care specifically through virtual reality remains an area for future exploration.

### Digital Technologies Detracting From Compassionate Care

In all, 3 studies identified in this review depicted digital technologies as detracting from compassionate care [39,43,55]. The articles that did discuss this aspect focused on the use of provider-based technologies (based on the WHO Classification of Digital Technologies), and primarily gathered information from the provider perspective. A greater understanding of provider and patient differences in their experiences and perceptions surrounding the role of digital technology in health care is necessary to fully understand the role of digital technologies in contributing to compassionate care in practice.

### Considerations for Digital Technology Implementation

Tables 6 and 7 present a summary of facilitators and barriers associated with each type of digital technology identified in this review. To our knowledge, this is the first review of its kind to appraise digital technologies in relation to compassionate care. Given the limited resources available at health system and organizational levels, investing in implementing a new digital technology can be a significantly resource-intensive undertaking. This summary can aid in the evaluation of digital technologies to ensure decision makers are investing in technologies that are aligned with organizational values and principles that relate to the provision of person-centered and compassionate care, and help to audit existing technologies in relation to delivering compassionate care.

### Limitations

Owing to the nature of scoping reviews, the quality of each identified article was not assessed. Although every effort was made to ensure all articles which may involve compassion in mental health care were included, the subjective nature of compassion may mean some articles were not captured in this review. As both compassion and the intersection between digital technologies and compassion are relatively understudied fields, the models leveraged in this review to classify the types of technologies identified may not be the appropriate taxonomies of organization. Grey literature was also omitted in this review.

### Future Steps

Ultimately, the successful use of digital technologies to facilitate compassionate mental health care requires health care organizations to invest the time and resources to leverage implementation science. In addition, health care professionals need to adapt to environmental and contextual factors to appropriately choose technologies to meet needs at the levels of patient, organizational, and population health needs. Future research should focus on expanded implementation of digital technologies in mental health care and identifying both technologies and specific settings where compassionate care would not be possible without digital technology. This information can then be used by digital technology developers and institutions to inform the creation and development of technologies that result in the best outcomes for both health care professionals and patients. Furthermore, identifying how to teach health care professionals to meaningfully use technologies in ways that convey compassionate care should be explored.

In addition, future knowledge translation plans include traditional techniques such as presenting at conferences and giving lectures to those practicing in the mental health field. Other plans include engaging practicing mental health professionals and students in a discussion about the topic to increase awareness of digital compassion.

### Conclusions

This review’s inquiry into the intersectionality between contemporary digital technology and compassion is a highly relevant and emerging topic, particularly in mental health care. The current state of digital technology in mental health care lends itself well to facilitate compassionate care delivery, particularly when used to serve patients who may not have had the chance to receive health care previously, or who may be uncomfortable or restricted in direct face-to-face interactions. Although there is still much to understand and uncover, health care organizations and professionals must consider the advantages and limitations of each type of digital technology for practice, particularly at this time where the discussion is only at its outset. As technology inevitably continues to diffuse throughout mental health care, these considerations alongside patient feedback will be instrumental to ensure that digital tools are, and continue to be, aligned with provider and patient needs. Ultimately, compassion and the integration of digital technology in mental health care should be seen as vital and complementary aspects of obtaining the best patient outcomes, as mediums to accentuate meaningful human connections rather than inanimate products of modern innovation.

## Appendix

Multimedia Appendix 1 Preferred Reporting Items for Systematic Review and Meta-Analysis—Scoping Review Checklist for Scoping Review.

## Abbreviations

AMS: Associated Medical Services
CBT: cognitive behavioral therapy
EHR: electronic health record
MEDLINE: Medical Literature Analysis and Retrieval System Online
PRISMA: Preferred Reporting Items for Systematic Review and Meta-Analysis
RQ: research question
WHO: World Health Organization
