# Supplementary material for: Delivery of Compassionate Mental Health Care in a Digital Technology–Driven Age: Scoping Review
Source: J Med Internet Res. 2020 Mar 6;22(3):e16263. doi: 10.2196/16263 (PMC7084292; doi:10.2196/16263)
Supplement: Multimedia Appendix 1 [file jmir_v22i3e16263_app1.docx]

| Supplementary Material. PRISMA-ScR Checklist | | | |
| --- | --- | --- | --- |
| **Section** | **Item** | **PRISMA-ScR Checklist Item** | **Reported On** |
| **Title** | 1 | Identify the report as a scoping review. | Page 1 |
| **Abstract** |  |  |  |
| Structured summary | 2 | Provide a structured summary that includes (as applicable) background, objectives, eligibility criteria, sources of evidence, charting methods, results, and conclusions that relate to the review questions and objectives. | Page 3-4 |
| **Introduction** |  |  |  |
| Rationale | 3 | Describe the rationale for the review in the context of what is already known. Explain why the review questions/objectives lend themselves to a scoping review approach. | Page 5-6 |
| Objectives | 4 | Provide an explicit statement of the questions and objectives being addressed with reference to their key elements (e.g. population or participants, concepts, and context) or other relevant key elements used to conceptualize the review questions and/or objectives. | Page 7 |
| **Methods** |  |  |  |
| Protocol and registration | 5 | Indicate whether a review protocol exists; state if and where it can be accessed (e.g. a Web address); and if available, provide registration information, including the registration number. | Page 7 |
| Eligibility Criteria | 6 | Specify characteristics of the sources of evidence used as eligibility criteria (e.g., years considered, language, and publication status), and provide a rationale | Page 10 |
| Information sources | 7 | Describe all information sources in the search (e.g., databases with dates of coverage and contact with authors to identify additional sources), as well as the date the most recent search was executed. | Page 10 |
| Search | 8 | Present the full electronic search strategy for at least 1 database, including any limits used, such that could be repeated. | Available in protocol |
| Selection of sources of evidence | 9 | State the process for selecting sources of evidence (i.e., screening and eligibility) included in the scoping review. | Page 10-11 |
| Data charting process | 10 | Describe the methods of charting data from the included sources of evidence (e.g. calibrated forms or forms that have been tested by the team before their use, and whether data charting was done independently or in duplicate) and any processes for obtaining and confirming data from investigators. | Page 11 |
| Data items | 11 | List and define all variables for which data were sought and any assumptions and simplifications made. | Page 11 |
| Critical appraisal of individual sources of evidence | 12 | If done, provide a rationale for conducting a critical appraisal of included sources of evidence; describe the methods used and how this information was used in any data synthesis (if appropriate). | Not applicable |
| Summary measures | 13 | Not applicable for scoping reviews. | Not applicable |
| Synthesis of results | 14 | Describe the methods of handling and summarizing the data that were charted. | Page 11 |
| Risk of bias across studies | 15 | Not applicable for scoping reviews. | Not applicable |
| Additional analyses | 16 | Not applicable for scoping reviews. | Not applicable |
| **Results** |  |  |  |
| Selection of sources of evidence | 17 | Give numbers of sources of evidence screened, assessed for eligibility, and included in the review, with reasons for exclusions at each stage, ideally using a flow diagram. | Page 13 |
| Characteristics of sources of evidence | 18 | For each source of evidence, present characteristics or which data were charted and provide the citations. | Page 14-15 |
| Critical appraisal within sources of evidence | 19 | If done, present data on critical appraisal of included sources of evidence (see item 12). | Not applicable |
| Results of individual sources of evidence | 20 | For each included source of evidence. Present the relevant data that were charted that relate to the review questions and objectives. | Page 12-26 |
| Synthesis of results | 21 | Summarize and/or present the charting results as they relate to the review questions and objectives | Page 12-26 |
| Risk of bias across studies | 22 | Not applicable for scoping reviews | Not applicable |
| Additional analyses | 23 | Not applicable for scoping reviews | Not applicable |
| **Discussion** |  |  |  |
| Summary of evidence | 24 | Summarize the main results (including an overview of concepts, themes. and types of evidence available). Link in the review questions and objectives, and consider the relevance to key groups. | Page 27-30 |
| Limitations | 25 | Discuss the limitations of the scoping review process. | Page 30 |
| Conclusions | 26 | Provide a general interpretation of the results with respect to the review questions and objectives, as well as potential implications and/or next steps. | Page 31 |
| Funding | 27 | Describe sources of funding for the included sources of evidence, as well as sources of funding for the scoping review. Describe the role of the funders of the scoping review. | Page 32 |
